# A Cancer Crusade

**Published:** 1920-04-10

**Authors:** 


### A CANCER CRUSADE.

### Many Facts and More Possibilities.

While other diseases may for a short time hold the stage and get more notice from the Press, there is no question that cancer exercises the greatest hold over the mind of the average man or woman. Other diseases may claim attention for a time; some with every reason, as syphilis, gonorrhœa, and tubercle, others less justifiably, as rabies and encephalitis lethargica, and a few with no justification whatever, as in the pellagra scare, which was so vigorously worked before the war. All these have their day, but cancer remains constant.

This being so, it may be of interest to plan an Anti-Cancer Crusade on the lines of the present Anti-Venereal Diseases Campaign, and to see what good might result from it.

One would start, of course, by saying that in the early stages cancer can be cured; this is true, and will well bear repeating. Then the next point would be that cancer, in its early stages, is not painful. These two points by themselves, if only they could be thoroughly instilled into every person, both in the medical profession and amongst the lay public, would greatly help to the more efficient treatment of this disease.

### Need for Early Examination.

Then, still to deal with generalities, the next point is that cancer is not contagious; this is true, and would bring a relief to many nervous persons. Next, that cancer is not hereditary; this is not absolutely true, though very nearly so, so nearly so that we would be quite justified in stating it for a fact. Then we should come to particular instances, and give advice which certainly ought to be common knowledge. The breast—any lump occurring in the breast should be examined by a doctor at once, and even if not considered to be malignant, should be kept under observation for many months. The propriety of advising the removal of small specimens for examination is open to debate, for it is more than probable that by such interference the formation of metastases is encouraged.

Any woman, who, after the climacteric, has what seems to be a return of her periods, or any sort of a discharge, no matter whether it be blood-stained, foul, or no, should at once submit to a thorough vaginal examination, and no secondary considerations should deter either her or her doctor. Though this is especially true of those past the climateric, it is also true of younger women, and unexplained menstrual irregularity or the appearance of a discharge should be the signal for a visit to the doctor and a thorough examination.

Then the rectum; all elderly persons who develop piles should have a rectal examination made lest the piles should be but a symptom of the underlying disease.

And lastly, any sores or cracks, lumps or ulcers, if they will not heal or tend to spread, and any warts, moles, or birth-marks, if they change in size, colour, or appearance, should be regarded as suspect, and should be examined by a doctor.

Here we have the basis of one pamphlet in our anti-cancer scheme, and it is impossible to deny that if simple information as that given above were distributed to all persons in all classes, and appreciated by them, the chances of patients coming for treatment with their disease still in a sufficiently localised condition to be capable of absolute removal, would be immensely increased.

The above leaves untouched, however, many sites of this disease. The diagnosis of some is so often only to be confirmed by operation, and their symptoms so vague, that more harm than good would result from sketching their early symptoms. To threaten every sufferer from indigestion with cancer of the stomach, and hold out laparotomy as the only certain means of diagnosis would bring the scheme very quickly into disrepute, but with the types mentioned above the case is very different.

### The Educational Factor.

That the widespread dissemination of such information would cause unnecessary alarm to many persons free from disease need not be counted as a drawback to the scheme. Already many thousands live in a constant state of dread lest they should be or become victims of this disease. In many cases their fears are quite ungrounded, and if, with a little suitable encouragement, they could be persuaded to see a doctor (and this, with the curability of cancer in the early stages as an incentive, should not be difficult), they would be enabled to lead lives free from care and constant worry. On the other hand, should their fears be just, they would stand a far better chance of cure if seen early, and at any rate would be no worse off.

Also the knowledge on the part of the doctor that his patients expected and desired a thorough examination would strengthen his position, and to a certain extent force his hand, and this, too, would be an advantage. It is an unfortunate fact, but none the less true, that some medical men will do all in their power to shirk examining their patients, especially when rectal or vaginal examination is in question.

### A Practical Opportunity.

A Government scheme with local cancer clinics, at which were available every help to the diagnosis of malignant disease, might follow, then the allotment of special cancer beds, or even the building of special hospitals to which patients could be admitted without the delay which is often unavoidable at present. And so a vast organisation would grow up, the final step no doubt being the formation of a research committee to investigate malignant disease in all its forms, and to prepare statistics from the scheme and its results in particular.

